# Taking back control of my mind

**DOI:** 10.1038/s41537-026-00743-0

**Published:** 2026-05-31

**Authors:** Jason Jepson

**Affiliations:** Schizophrenia, https://www.nature.com/npj

**Keywords:** Schizophrenia, Human behaviour

During the worst times, the voices I heard in my head were spoken by people I knew in real life or in my recent past. It was one of the biggest lessons to learn that the people with whom I was conversing could influence my actions, which was actually a mental illness. It was not real, but unreal. The unreal was based on real people whose actions could only be seen by me.

Even the people I loved had an unreal side that I thought only I could see. The figures of my past that I thought I rejected could make me second-guess myself as if they were in the same room. It took a while for me to realize that my people in my past did not think of me as much as I thought of them. In reality, they had gone on with their lives, yet I thought they were talking to me in my mind.

I remember being committed to the psych ward; however, I was in handcuffs. The psych ward was far away from my parents’ house, which was where I was living at the time. I remember telling my parents that I would start to take my medication when my V-A benefits came through. I did not know how crucial it was for me to be on medication.

The police were called to our home. They did their job of protecting me from harming myself or others, and they treated me very respectfully. I remember that in my nervousness, I even told them a joke, and they laughed. I did not fight them.

At the time, I did not know what was real inside my head and what was not real, but I did know I was tired of dealing with my issues. When the police came, I did not run or fight them. I thought I had no options left. I thought if I were ever going to get help with my issues, this would be how it starts. I actually slept in the back seat of the police car on the way to the hospital.

The police took off the handcuffs as a nurse interviewed me. At the time, I did not think I had schizophrenia. I even explained to her what I thought I had going on.

“When people make eye contact with me, they can hear me through telepathy.” I thought these were my special powers.

The psych ward was attached to a prison, and I met a man who was going to go back to prison after his stay in the psych ward. He told me that the psych ward’s food was better than prison’s food. Then I thought, no matter how far down I get, I will never go to prison. The psych ward was there for people to feel better. There was an open schedule. They told us when to eat and when to take our medication. This was a low point for most people. Here, I figured the only thing I could do was move up. I did what I was supposed to do, took my meds, and ate the food which was bad but not as bad as the prison.

After I was discharged from the psych ward, I was put in a homeless respite. My parents thought that was the safest place for me because they had never dealt with a schizophrenic before. I saw it as the next step of my journey. I did not know what recovery meant.

At the homeless respite, they fed us, and they gave us our medications at the same time each day. There was also a TV with a DVD player so we could watch movies. An employee who worked there even brought movies for us to watch. The respite was better than the psych ward because I could smoke whenever I wanted.

The homeless respite was where my meds really started working, and it was a hard lesson, but I realized I had schizophrenia. The chaos in my mind was not supposed to be there. Again, there was some structure, such as we could not sleep the day away. But most importantly, I did not feel judgment.

I was always simple, as it did not take much to make me happy. After I was discharged from the homeless respite, a mental health organization put me in a furnished room. All I needed was cigarettes and a TV. With this situation, there was much more freedom. I did not fight anybody in regard to taking my meds. I understood I had to take them for the rest of my life.

I could also come and go as much as I pleased. Sometimes my parents brought me back to their house for a day or two. I understood now why they felt the need to call the police. I could not imagine calling the police on someone you loved. They had to do it, and it was the only way I would have gotten help and started taking my medication. During the whole journey, they did what they could for me. I owe them a lot for that hard decision they had to make.

I was at my parents’ house when my dad told me my veteran’s benefits had come through. It had been a year since I began that process. A long, tedious year, I was told I was lucky that it only took a year. I was not rich, but I was financially secure. My dad would be in charge of my money. I did not have a problem with that. Money can be stressful, and I trusted my dad.

I was diagnosed with schizoaffective disorder, bipolar type. This diagnosis has both schizophrenia and bipolar depression. Several years later, I was with my psychiatrist. I told her about some suicide ideation I was having at the time. She looked at my profile and saw I had never taken anything for my depression. I had dealt with the schizophrenia part of the diagnosis, but now I was going to take a “happy” pill for my depression.

Today I have my own apartment, and I pay rent. It is just me in the apartment. I have plenty of food to eat, clothes to wear, and my own schedule. I value all the experiences I have gone through in my mental health journey. It could have gone a lot differently, but I am here with the help of my parents. They never gave up, and neither did I. I love that I live an independent life. With the help of my therapists, I have produced strategies to cope with my symptoms when they occur. I keep my daily routine structured, and I stay as active as possible. Yes, there have been some unbelievably bad days, but I look back and know that I have learned something about myself at every step.

Now I have had my schizophrenia long enough that I know the difference between an unreal voice and what is the actual voice that is talking to me. When I am by myself in my apartment, it is easy. I can conclude that the voice I am hearing is part of my schizophrenia. If I am in a group, I try to realize if the voice I hear belongs to one of the individuals I am sitting with. I eventually won the battle of mental chaos by taking on the war of schizophrenia. With the combination of medication and a faithful support team, I can manage control of schizophrenia symptoms.

God had not given up on me, and my parents had not given up on me. This was the beginning of a journey known as recovery. Schizophrenia had lost, and I had everything to gain in my life. I am still in the midst of recovery from schizophrenia. I will not revisit the chaos in my mind again. I experienced a period of instability, but I regained control after accepting the help that was being offered to me.

